# Estimating above-ground biomass of subtropical forest using airborne LiDAR in Hong Kong

**DOI:** 10.1038/s41598-021-81267-8

**Published:** 2021-01-18

**Authors:** Evian Pui Yan Chan, Tung Fung, Frankie Kwan Kit Wong

**Affiliations:** 1 Institute of Future Cities, The Chinese University of Hong Kong, Hong Kong, China; 2 Department of Geography and Resource Management, The Chinese University of Hong Kong, Hong Kong, China

**Keywords:** Ecosystem services, Ecology, Environmental sciences, Environmental social sciences

## Abstract

Seventy-percent of the terrestrial area of Hong Kong is covered by vegetation and 40% is protected as the Country Park. The above-ground biomass (AGB) acts as reliable source of carbon sink and while Hong Kong has recognized the importance of carbon sink in forest and urged for forest protection in the latest strategic plan, yet no study has been conducted on assessing the baseline of terrestrial AGB and its carbon storage. This study compared and estimated the AGB by the traditional allometric modeling and the Light Detection and Ranging (LiDAR) plot metrics at plot-level in a subtropical forest of Hong Kong. The study has tested five allometric models which were developed from pantropical regions, subtropical areas and locally. The best model was then selected as the dependent variable to develop the LiDAR-derived AGB model. The raw LiDAR point cloud was pre-processed to normalized height point cloud and hence generating the LiDAR metric as independent variables for the model development. Regression models were used to estimate AGB at various plot sizes (i.e., in 10-m, 5-m and 2.5-m radius). The models were then evaluated statistically and validated by bootstrapping and leave-one-out cross validation (LOOCV). The results indicated the LiDAR metric derived from larger plot size outperformed the smaller plot size, with model R^2^ of 0.864 and root-mean-square-error (RMSE) of 37.75 kg/ha. It also found that pantropical model was comparable to a site-specific model when including the bioclimatic variable in subtropical forests. This study provides the approach for delineating the baseline of terrestrial above-ground biomass and carbon stock in subtropical forests upon an appropriate plot size is being deployed.

## Introduction

Forest biomass plays an important role in balancing the carbon cycle as it accounts for 45% of the terrestrial carbon pool and 31% of total carbon sink^1^_._ AGB itself, accounts for 70% to 90% of total forest biomass and thus taking up around 30% of terrestrial carbon^2^. Hong Kong, as a city, with 70% of land covered by vegetation, has recently formulated the first city-level Biodiversity Strategy and Action Plan (BSAP); and one of the action plans is to identify the ecosystem services as to understand the values of our habitats^3^. An important service provided by the forest is Carbon Storage and Sequestration yet it will not be estimated under the assessment plan in near future. Climate change has affected around 68.5 million of world population and has brought up to $131.7 billion of economic losses^4,5^, which explained the importance of accounting the carbon storage globally. To mitigate the effect climate change, one of the latest technologies that prevent carbon dioxide (CO_2_) from entering the atmosphere is through Carbon Sequestration, Storage and Utilization (CSSU), by means of carbon uptake technologies and carbon dioxide separation^5^. CCSU is a bundle of technologies that captures over 90% of the carbon being emitted from burning of fossil fuels for energy uses^6^. The stored carbon could be transport to the nearly-depleted oil and gas fields and hence to replenish the oil and gas projection; or use CO_2_ to enhance oil, gas and coal bed methane recovery; or offshore and onshore deep saline formations. The main technologies of CCSU are pre-combustion, oxyfuel combustion and post-combustion technologies^4^.

While modern technologies may help mitigating climate change, the natural carbon pools should not be overlooked. According to Le Quéré et al.^1^, the Global Carbon Budget 2018, around 23% of carbon was stored in the ocean and 31% was stored in the biosphere (i.e., terrestrial carbon pool) averaged over the last decade. Terrestrial carbon pool, which serves as a reservoir of carbon that has the capacity to accumulate or release carbon, around one-third of the terrestrial carbon was stored by the biomass (including AGB and below-ground biomass (BGB); almost two-third of it was stored in the soil and the remaining was captured by the dead organic matter^2,7^. Amongst, AGB is the most dynamic and fluctuating^8–10^. Therefore, to quantify biomass as for carbon sequestration assessment, estimating AGB is a priority task.

Despite the fact that harvesting vegetation is the most accurate method to determine the biomass content, it is time consuming, illegal, if not impractical^11,12^ to be conducted extensively. Other than destructive sampling method, plot-level biomass can be estimated by interpolation or extrapolation over the study area by indirect and non-destructive methods^11,13^, such as empirically based on allometric equations developed by destructive samples that enable us to estimate biomass by some easily measurable variables (e.g. diameter-at-breast height (DBH) and height (H)^14–16^.

Allometric modeling is the most common approach applied by the forestry scientists and ecologists in biomass estimation. The law of simple allometry, is conceived as how the properties of an organism change (e.g., DBH, height, and wood density (ρ)) in relation to proportional change in size-related traits (i.e., body size, biomass)^17^. This allometric relationship can be expressed in a power-law form:

1 $${AGB}_{i}=a{X}_{i}^{b}+ {\varepsilon }_{i},$$

where ${AGB}_{i}$ is above-ground biomass of the tree and *X* is the value of a trait for the *i*th tree being sampled; such as the height (in m) or DBH (in cm), *a* and *b* are model parameters and ${\varepsilon }_{i}$ is the error of the measured *i*th tree^18^; the logarithmic form as shown below is more prevalent due to its simplicity and linearity^17,19,20^:

2 $$\text{ln}\left(AGB\right)={\upalpha }+\beta \text{ln}{((DBH)}^{2}H\rho ).$$

Towards the end of 1980s, Brown et al.^19^ developed regression equations and applied to 5300 trees from 43 independent sample plots, to estimate total AGB as a function of DBH, height and Holdridge life zone group (Wet/Moist/Dry)^21^. Subsequently, Chave et al.^15^ conducted a reassessment and evaluated the robustness of AGB models across tropical forests, which indicated that diameter, wood density, height and forest type are of decreasing order of importance in estimating biomass. It contributed significantly to forest carbon accounting and being applied in the United Nations ‘Reducing emissions from deforestation and degradation (REDD)’ programme^16^. More recently, Chave et al.^16^ proposed an allometric model which could be applied across pantropical regions, by using the form factor, “ρ(DBH)^2^H”, or Bioclimatic variable (E), to derive the relationship between DBH and height when height is unavailable. However, this equation has not been tested extensively in subtropical moist forests.

The most precise method for AGB estimation is to build the empirical and species-specific model. However, this is more appropriate to forests with homogenous species such as the temperate forests or plantations^22^. In the natural tropical and subtropical forests, one may have over 300 tree species thriving within one hectare (ha) of forests. In that sense, a mixed-species modeling approach would be more practical. Various mixed-species models were developed in pantropical or tropical regions^15,16,23^.

In the traditional field measurement method, the estimation is restricted by the limited spatial extent of forest inventories due to high time and labor cost^12,24^. Remote sensing technology can fill the gaps by capturing data covering a comparatively large spatial extent at a relatively quick and cost-effective way^24^. Fundamentally, the remote sensing-based AGB estimation methods assume biomass is highly correlated with certain forest characteristics captured by the sensors^12,25–27^. Apart from Optical and RADAR data, LiDAR is the latest technology and treated as a practical alternative to traditional field surveys on AGB; given its ability to provide detailed three-dimensional vegetation structure which is useful to derive biomass-related parameters, by retrieving the vertical distribution of ‘laser canopy heights’ and that of ‘forest canopies (leaf area)’ measured from field measurement^28^.

LiDAR has a strong potential in estimating forest biomass and volumes across AGB ranges and has been found to have significant correlations with biomass in forested ecosystems^12,29,30^. The common LiDAR plot metrics derived from the point clouds can be grouped into four categories: descriptive, height percentile, intensity and canopy cover. Height percentiles, which describe the canopy height distributions within the plot, were suggested to be an estimator of AGB and its carbon content^31,32^ based on the principle that different levels of LiDAR beams penetrating into the forest canopies delineate the canopy structural characteristics. In plot-level estimation, the selection of appropriate plot size in deriving the plot metrics is influential to model accuracy, as an optimal plot size reduces the co-registration error and the edge effect^33,34^.

Airborne LiDAR had been utilized to estimate stands in boreal and temperate forests since 1990s^29,35–37^. Recently, there are more applications of LiDAR in estimating biomass and carbon content in tropical regions^38,39^, particularly in subtropical forests^39^, though its potential is not yet fully realized and evaluated. Table 1 compares the three most common AGB estimation methods (i.e., Direct Harvesting, Allometric Modeling and LiDAR Modeling).

**Table 1:** Comparison table on the common AGB estimation methods.

	Direct harvesting	Allometric modeling	LiDAR-derived AGB modeling
Sampling method	Direct	Indirect	Indirect
	Destructive	Non-destructive	Non-destructive
Fundamental principles on AGB estimation	Based on field and laboratory measurement	Based on the relationship with measurable tree variables (e.g., DBH, height)	Based on LiDAR metrics derived from the LiDAR point clouds
	Ratio of dry weight and fresh weight^10,14,40^	Law of simple allometry, how trees’ properties (e.g. biomass) scale allometrically with body size^17,19^	The relationship between the vertical distribution of ‘Laser canopy heights’ and the vertical distribution of ‘Forest canopies’^24,28,31,41,42^

In view of this, the main objective of the study is to estimate AGB by both the approaches of allometric models (i.e., pantropical, subtropical and local) and LiDAR-derived metrics; to compare them by applying both onto the same subtropical forest plot in Hong Kong. An optimal model form and plot size are to be proposed and discussed. Upon the completion of study, it aims to generalize an AGB estimation model by utilizing the LiDAR technology, to encourage a periodic assessment of the terrestrial biomass in forest of Hong Kong. The study expects to arouse the awareness of policy makers and the public on the importance of terrestrial biomass and its carbon storage and sequestration ecosystem services.

## Materials and methods

### Study area

**Figure 1:**
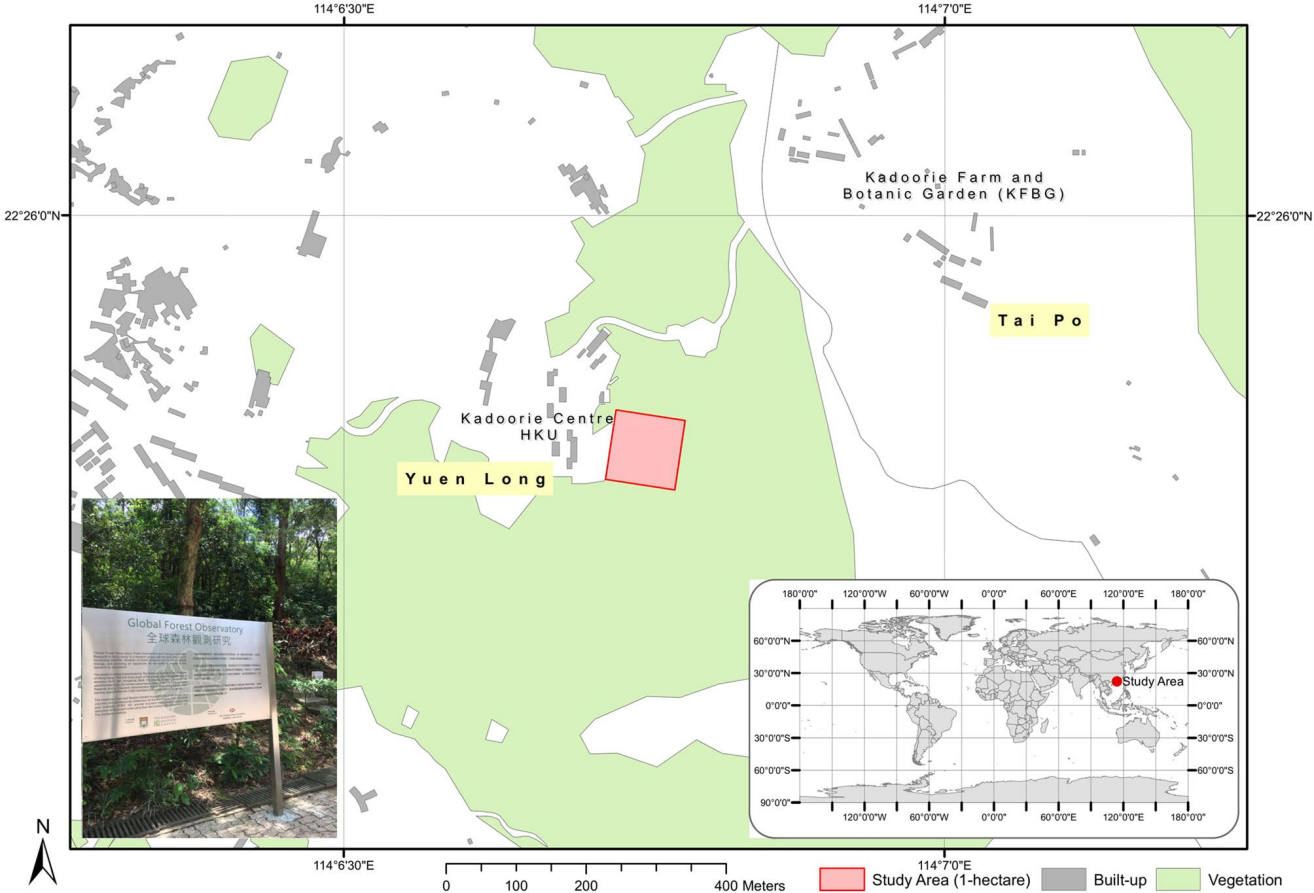
The study area: 1 hectare subtropical moist young forest plot, a full demonstration plot of the Forest Global Earth observatory (ForestGEO) project (https://forestgeo.si.edu/), located in Hong Kong in understanding the long-term forest dynamic. The map is generated by Authors using ArcMap version 10.5 (https://desktop.arcgis.com/en/arcmap/).

The study area is a one-hectare (ha) subtropical mixed young forest in the age of 20 to 30 years of Hong Kong. It is located at Shek Kong (22.428774, 114.114968), in between the Kadoorie Farm and Botanic Garden (KFBG) and Kadoorie Institute, The University of Hong Kong (HKU) as shown in Fig. 1. This is a forest dynamic plot under ‘The Center for Tropical Forest Science–Forest Global Earth Observatory (CTFS-ForestGEO) (https://forestgeo.si.edu/). It acts as a research base on the forest dynamics, forest biodiversity, carbon sequestration and more, which also provide opportunity for public involvement in scientific research.

The one-hectare forest plot was demarcated by 25 quadrats in 20 m × 20 m each, which were further sub-divided into sixteen 5 m × 5 m sub-quadrats; delineated with permanent marker poles by the professional surveying team. The forest survey was launched on January 11th, 2012 and completed on September 6th, 2012. A total of 63 species, 10,442 individual trees with 20,888 stems were recorded in the site. The stem locations (UTM WGS84 coordinate system) were recorded in nearest 5 cm, and DBH were measured at 1.3 m at breast height in nearest 1 mm. The dominant species are *Litsea rotundifolia*, *Psychotria asiatica, Ilex asprella* and *Aporosa dioica*, which all are native species and accounted for over 80% of stems with the study area. The forest condition of the study area is shown in Table 2.

**Table 2:** The forest condition and descriptive statistics.

Forest type	Subtropical mixed young forest
Age	20–30 years
Diameter range (cm)	1–57 (mean: 25.5, standard deviation (S.D.): 23.8)
Number of stems	20,888
Stem density (per m^2^)	2.1/m^2^
Species composition	63
Tree-to-shrub ratio (%)	48:52
Topography	Gently flat, from 218 to 257 m above sea level (a.s.l.)

### Methods

**Figure 2:**
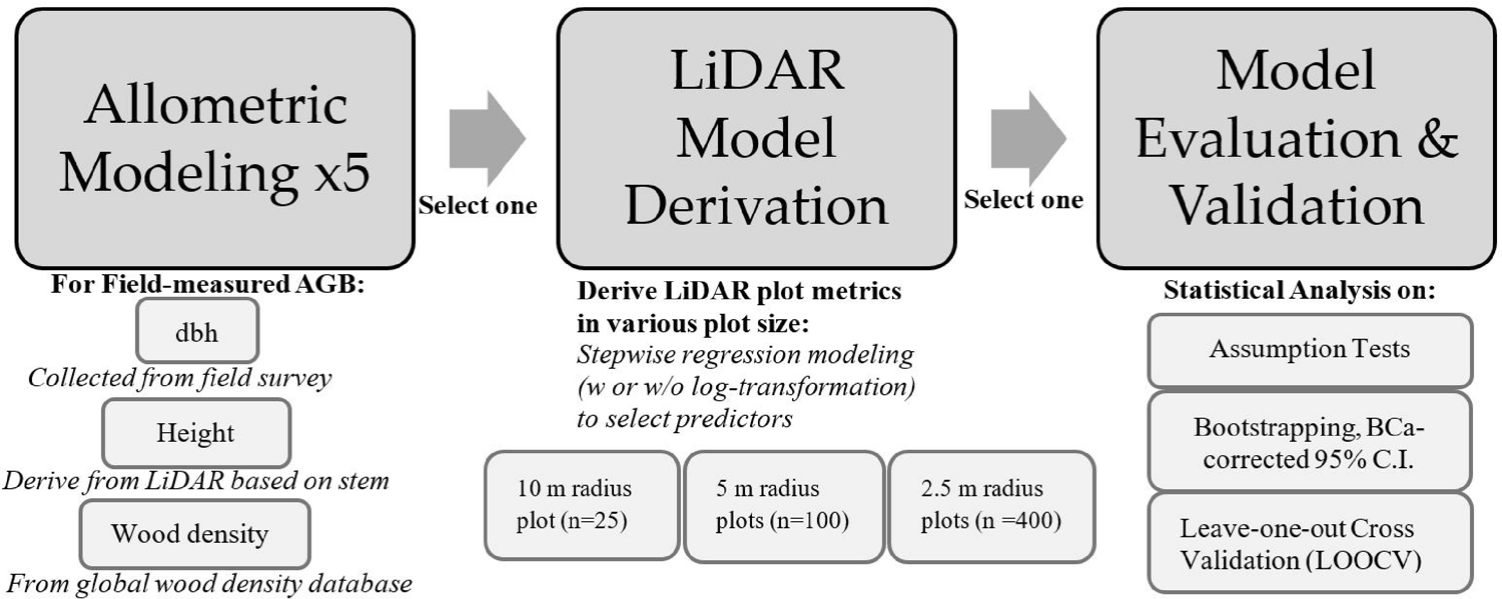
The workflow of the study. Stage 1: allometric modeling to compute ground-truth AGB. Five allometric models were compared and the best one is selected to calibrate model in the next stage. Stage 2: LiDAR AGB Model Derivation, by comparing LiDAR plot metrics derived in different plot size (i.e. 10 m, 5 m, 2.5 m radius). Stage 3: Model Evaluation & Validation on the best model selected from Stage 2 (Source: Authors).

**Figure 3:**
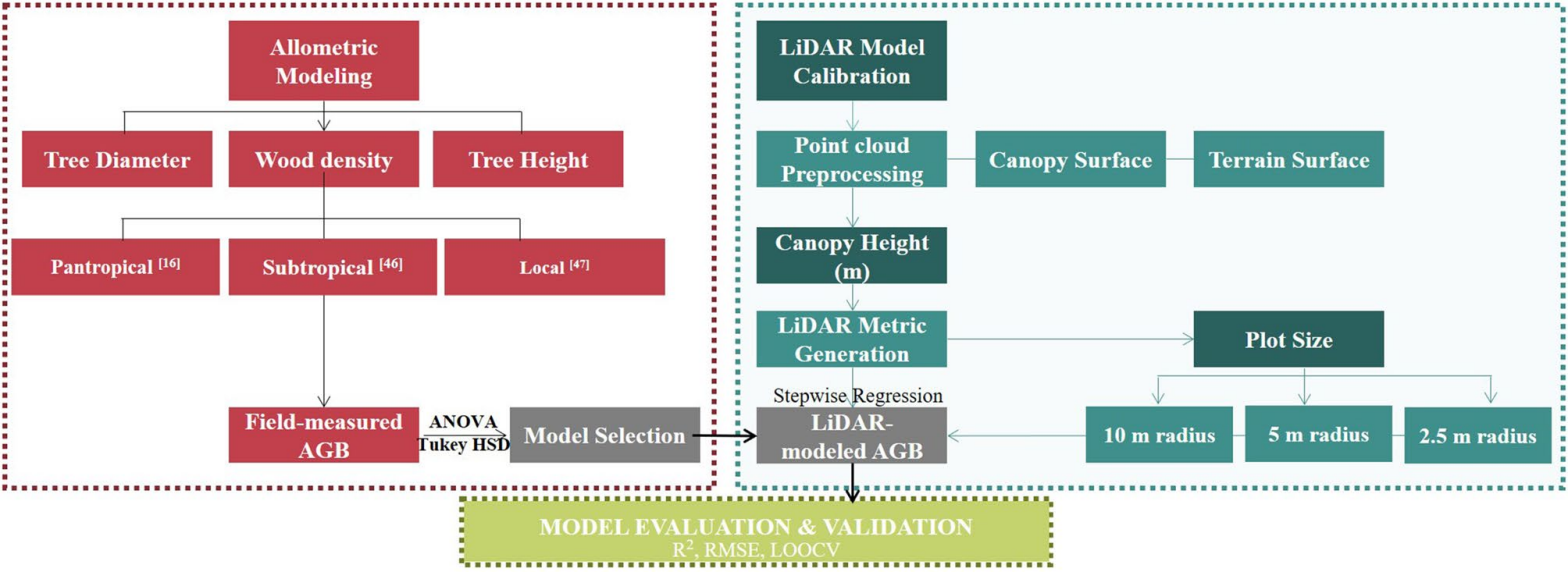
The graphical abstract of the study (Source: Authors).

A three-stage methodological framework was outlined for this study as shown in Figs. 2 and 3. In the first stage, parameters including DBH, wood density and stem location were recorded for field-measured AGB computation. DBH was directly obtained from site, wood density (in g cm^−3^) was obtained from the global wood density database^43,44^ or World Agroforestry database^45^ (http://db.worldagroforestry.org//wd), up to species level. If the species was not recorded in the database, the value was replaced by its genus averaged wood density value. Tree height was derived from the LiDAR data with reference to the recorded x,y location of the stems. The second stage is LiDAR AGB model derivation. Five allometric models (Model 1 to Model 5) were used to estimate the AGB of individual trees based on field-measured parameters. The allometric model with the lowest model error was selected to compute the ‘Field-measured AGB’, which would be used as the dependent variable to develop the LiDAR-derived AGB model. The LiDAR plots metrics were generated in various plot-size (i.e., 10 m radius, 5 m radius and 2.5 m radius) within the study area. Stepwise linear regression was used to select the important and significant predictors in each regression model. Three different model forms were tested (Model I, II, III as discussed in “Stage 2: LiDAR AGB model derivation” section), The LiDAR derived AGB regression models would be evaluated by means of assumption tests and bootstrapping; as well as cross-validation (CV) in the last stage of model evaluation and validation.

#### Stage 1: allometric modeling

20,888 stems from 63 species, were planned to be used to develop the allometric models. Amongst, the 81% of the wood density value were measured up to species level (i.e., 51 species) and 19% were up to genus level (i.e., 12 species). However, as the tree height parameter was retrieved from the LiDAR 1 m Canopy Height Model (CHM), 263 stems found no value and thus the sample size reduced to 20,625 stems. The DBH of stems ranged from 1.0 cm to 57.1 cm, with mean of 2.55 cm and S.D. of 2.38 cm.

The selected five allometric models included two pantropical models by Chave et al.^16^; two subtropical models by Xu et al.^46^ and one local model by Nichol and Sarker^47^ were assessed. The inclusion of the subtropical models from Xu et al.^46^ is because of the similar forest type (i.e., subtropical moist forest from southern China) with our study area; whereas the pantropical models from Chave et al.^16^ is due to its large database across the pantropical regions yet not being explored adequately its applicability in subtropical forests.

The five allometric models were compared by One-way ANOVA and the Tukey HSD post-hoc analysis^48^. Model 1 (Eq. (3)) was the best model proposed by Chave et al.^16^, comprised of wood density (ρ), diameter-at-breast height (DBH) and height (H) as shown below (AIC = 3130):

3 $$AGB=0.0673\times {\left(\rho {(DBH)}^{2}H\right)}^{0.976}$$

However, it is very unlikely to have a precise tree height data in a closed canopy^49^. Therefore, an alternative allometric equation without height was then proposed by Chave et al.^16^ and adopted as Model 2 (Eq. (4)) in our study (AIC =  − 4293):to represent the subtropical AGB

4 $$AGB=exp\left[-1.803-0.976E+0.976\text{ln}\left(\rho \right)+2.673\text{ln}\left(DBH\right)-0.0299\left[{(\text{ln}(DBH))}^{2}\right]\right]$$

The bioclimatic variable, “E”, which made up of three parameters to account for climatic variations: Temperature seasonality (TS), Long-term Maximum Climatological Water Deficit (CWD) and Precipitation Seasonality (PS). The formula of the bioclimatic variable, “E”, is shown below (Eq. (5)):

5 $$E={\left(0.178 \times TS-0.938 \times CWD-6.61 \times PS\right)}^{{10}^{-3}}$$

The monthly mean temperature (℃), precipitation (mm) and evapotranspiration (mm) data were obtained from the Hong Kong Observatory^50^ in the period of 1981 to 2010 to compute the three variables. E was then computed as 0.261 and was input into Eq. (4) to derive the ground-truth AGB of individual stems.

The AGB models developed by Xu et al.^46^ were conducted in the subtropical mixed-species moist forest in southern part of China. Two allometric models from the study were selected to represent the subtropical AGB model. Model 3 (Eq. (6)) assumed tree height is available (by extracting from LiDAR):

6 $$AGB=\text{exp}(-2.334+2.118\text{ln}(DBH)+0.5436\text{ln}(H)+0.5953\text{ln}(\rho ))$$

Meanwhile, an alternative allometric model would be used by assuming tree height was not available, which is the Model 4 (Eq. (7)) of this study:

7 $$AGB=\text{exp}(-1.8226+2.4105\text{ln}\left(DBH\right)+0.5781\text{ln}(\rho ))$$

Nichol and Sarker^47^ developed a local allometric model for Hong Kong by harvesting 75 trees from 15 dominant species of Hong Kong. DBH and H within 50 circular sample plots from a variety of tree stands were measured, which were then used to establish allometric model with field measured AGB. The best allometric model was a model using DBH as the sole parameter and thus selected as the Model 5 (Eq. (8)) of this study:

8 $$AGB=\text{exp}(-1.8226+2.4105\text{ln}\left(DBH\right)+0.5781\text{ln}(\rho ))$$

#### Stage 2: LiDAR AGB model derivation

The airborne LiDAR data used in this study was captured by Optech Gemini ALTM Airborne Laser Terrain Mapper and acquired by The Government of the Hong Kong Special Administrative Region from December 1st, 2010 to January 8th, 2011. A total of 5575 ground truth points were generated, with horizontal accuracy of 0.294 m (95% confidence interval (C.I.)). Vertical accuracy was also assessed against the orthometric heights of Hong Kong Principal Datum (HKPD), the average vertical accuracy was 0.1 m (95% C.I.). Multiple returns were recorded per pulse up to four range measurements (i.e., first, second third and last). The LiDAR acquisition parameters are shown in Table 3.

**Table 3:** The LiDAR acquisition parameters.

Scan frequency	47 Hz
Flight speed	120 knots
Flight time (laser time)	60 h (21 h)
Field of view	0 ± 20°
Average point spacing	0.336 m
Average point density	8.86 per m^2^
Acquisition dates	December 1, 2010 to January 8, 2011

Prior to generation of the plot metrics, the LiDAR data was pre-processed by creating the ground TIN by extracting only the ground returns. The extraction and processing were performed in ArcMap 10.5 with LAStools extension (https://rapidlasso.com/lastools/) and the FUSION 3.7 (http://forsys.cfr.washington.edu/fusion/fusionlatest.html). The canopy surface was defined using all non-ground returns with height above 2.0 m. The reason of choosing 2.0 m as the threshold was to avoid canopy returns to be mixed with ground returns^51^. Moreover, ‘all returns’ were used instead of the ‘first returns’, since the former provided more information on the lower canopies or understory^51^, while over 50% of returned points in our study area were classified as medium or low vegetation. The ground TIN was subtracted from the canopy surface to compute the normalized height value of each canopy point. The LiDAR metrics were then derived from the normalized height point cloud.

**Figure 4:**
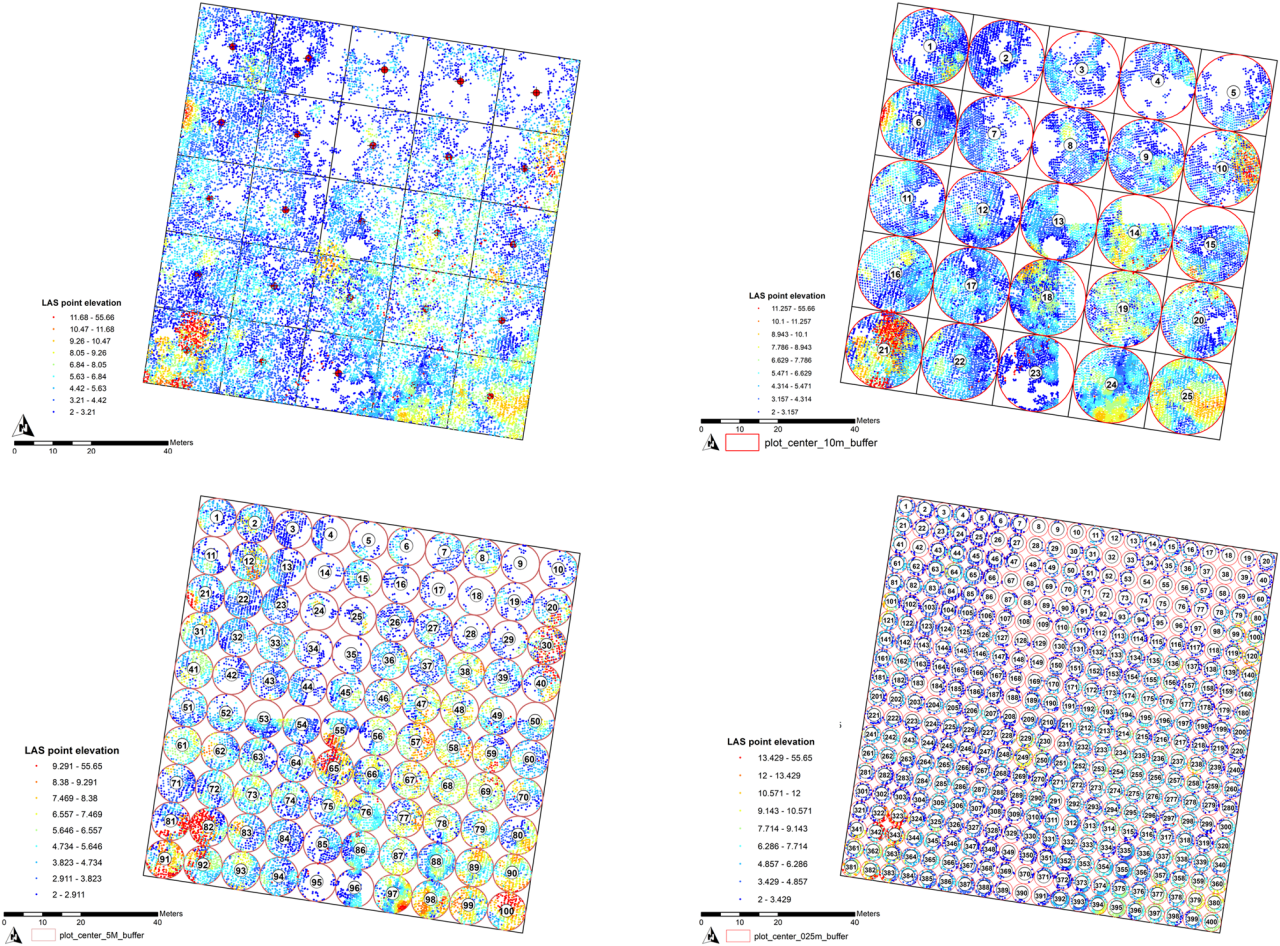
The (**a**) 1 ha rectangular plot clipped into (**b**) twenty-five 10 m radius circular plots; (**c**) one-hundred 5 m radius plots; and (**d**) four-hundred 2.5 m radius plots respectively; for the LiDAR metrics derivation. The maps are generated by Authors using ArcMap version 10.5 (https://desktop.arcgis.com/en/arcmap/).

To be compatible with the LiDAR dataset and to facilitate the plot delineation procedure, the entire 1 ha study area was divided into circular plots with three plot sizes: (1) twenty-five 10 m radius plots (16,182 stems), (2) one-hundred 5 m radius plot (15,538 stems) and (3) four-hundred 2.5 m radius plots (15,100 stems) as shown in Fig. 4a–d. Circular plots were considered more favorable than rectangular or square plots, since the periphery-to-area ratio was the smallest and thus minimized the number of edge trees^52^.

The 59 plot metrics, under the descriptive, height, intensity and canopy cover categories, were derived by the ‘cloudmetrics’ function in FUSION version 3.7. The LiDAR metrics, and its log-transformed metrics were input into stepwise regression model as independent predictors of AGB. Significant predictors were selected (F < 0.5 were entered and F > 1.0 were removed) into the regression model. Three sets of regression models were generated (Table 4) and the allometric models tended to be linear and normal after logarithmic transformation^18,19^.

**Table 4:** The input variables for the three regression models.

Model I		Model II		Model III	
Dependent variable (DV)	Independent variable (IV)	Dependent variable (DV)	Independent variable (IV)	Dependent variable (DV)	Independent variable (IV)
Raw	Raw	Log-transformed	Raw	Log-transformed	Log-transformed

**Figure 5:**
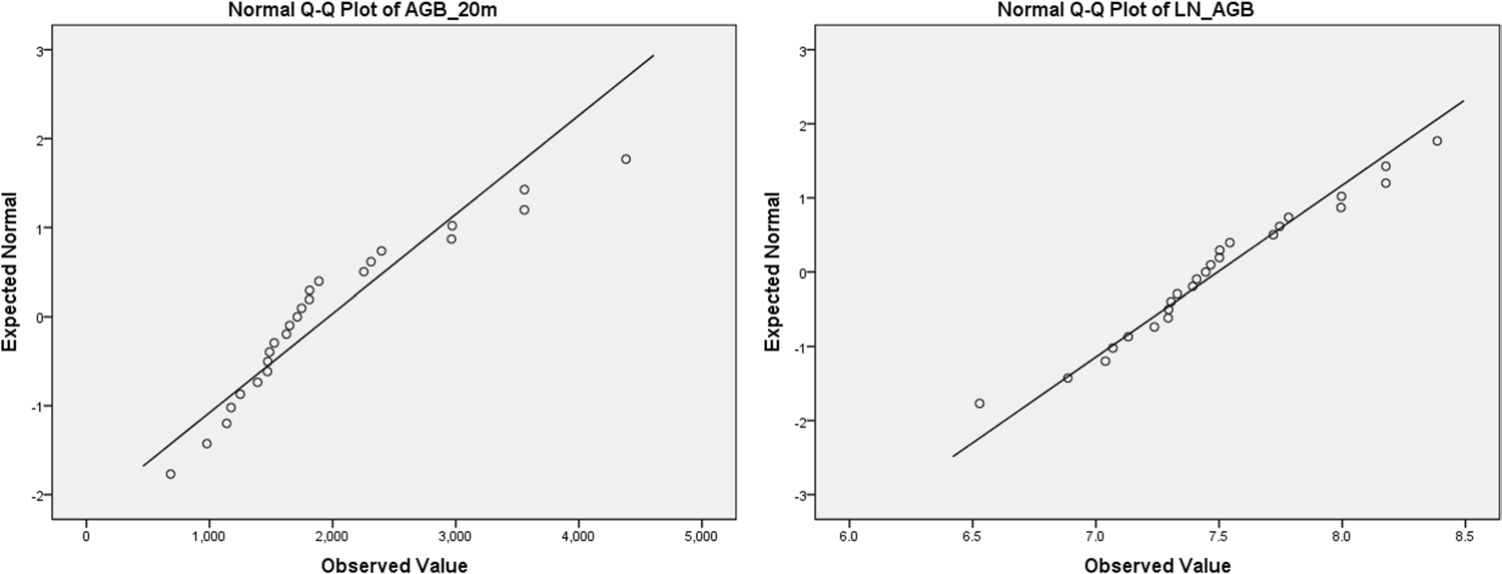
The normal Q–Q plot, in 10 m radius plot size, of (**a**) raw AGB and (**b**) log-AGB. After logarithmic transformation, the AGB (dependent variable) was normalized.

Observing the normal Q–Q plot (Fig. 5a) of the dependent variable (i.e., AGB) and the Kolmogorov–Smirnov statistic (d = 0.216, p < 0.05), it indicated the raw AGB (i.e., Model I) was non-normal. Therefore, AGB was then log-transformed into the log-unit (i.e., Model II); the Kolmogorov–Smirnov statistic (d = 0.135, p > 0.200) indicated a normal distribution (Fig. 5b) and which became the Model II. The further log-transformation into Model III did not indicate significant improvement and thus Model II in various plot sizes were to be further explored.

Assumption tests including test on normality, homoscedasticity and absence of multi-collinearity were conducted. The Normal P–P plot, scatter plots on residuals, ‘Tolerance’ index and Variance Inflation Factor (VIF) were applied to detect the violation of assumptions. If the ‘Tolerance’ index is smaller than 0.1, or VIF is greater than 10, it indicates a significance chance of collinearity^53^.

#### Stage 3: model evaluation and validation

AGB regression models were to be evaluated in this stage. The Model R^2^, Adjusted R^2^, Mean-absolute-deviation (MAE) and Root-mean-squared-error (RMSE) were reported to indicate the explanatory power of the model. R^2^ showed the amount of explained variance by the model, MAE was the average magnitude of error without considering the direction (Eq. (9)). RMSE (Eq. (10)) was the square root of the averaged squared residual and being sensitive to outliers (i.e., larger error) as the errors are squared. The RMSE would be reported in the unit of kg/ha.

9 $$MAE =\frac{\sum_{i=1}^{n}\left|(\widehat{y}-y)\right|}{n}$$

10 $$RMSE= \sqrt[]{\frac{{{\sum }_{i=1}^{n}(\widehat{y}-y)}^{2}}{n}}$$

$\widehat{\text{y}}$ is the *i*th estimate for AGB of each plot, y is the *i*th observation of that plot, divided by the $n$, denoting the sample size.

However, as the regression model was built with limited sample plots, it might subject to model overfitting. Bootstrapping was adopted to assess statistical accuracy in terms of the confidence intervals^54^. Ultimately, it provides a robust estimation of the standard errors, confidence intervals for estimates, including the model regression coefficient, mean and correlation coefficients. The model uncertainty of this study was assessed by 1000 runs of bootstrapping. The 95% “Bias-corrected and accelerated (BCa) confidence interval (C.I.)” on the beta coefficient of model predictors was reported.

Leave-one-out cross validation (LOOCV) was utilized for model validation. The model would be trained by all data points except the one being left out for validation^55^. The Predicted Residual Error Sum of Squares, PRESS, was the sum of the ‘squared deleted residuals’ (SSDR) of the *n−1* observation. Predicted R^2^ was computed by dividing PRESS by the total sum of square residual (SSTO) expressed in Eq. (11). The RMSE of the CV model was also reported as Eq. (12). The RMSE of the CV model without overfitting shall be approximate to that of the original model.

11 $${R}_{pred}^{2}=1-\frac{PRESS}{SSTO}$$

12 $$C{V}_{RMSE}= \sqrt[]{\frac{PRESS}{d.f.}},$$

*d.f*. stands for degree of freedom (i.e., *d.f.* = 21). These model calibration and validation work were conducted in IBM SPSS Statistics version 24.

## Results

### Stage 1: allometric modeling

The five proposed models were compared; based on the assumption that the local model was the optimal model potentially since it was a site-specific model built with local destructive samples. However, the model was subjected to bias and uncertainty as the species and tree diameter ranges were narrower when compared to the study area (Table 5). For that matter, we compared the local model with the other four non-local allometric models, to evaluate if any statistical significance can be detected and to find out a better alternative to the local model (Model 5).

**Table 5:** The species count, DBH and AGB range within the study area, as assessed by the pantropical and local allometric models; compared to the field survey data.

Model	Species count	DBH range (cm)	AGB range (kg)
Pantropical 2 [Model 2]	1125	5–212	1.23–76,060
Local [Model 5]	18	4.5–24.2	2–210
The study area	63	1–57.1	0.05–2120.78

The local model (Model 5) was built by the 15 dominant species in Hong Kong, with R^2^ of 0.933, RMSE of 13.525 kg. It was expected to be applicable to our subtropical mixed forest. However, wood density was being suggested to be an important predictor on AGB and capable to reduce model uncertainty^23,56^, yet the local model only depended on diameter to be the sole predictor on AGB.

There was significant difference among the five models statistically according to the ANOVA results. From the analysis of the Tukey Post-Hoc test as shown in Table 6, the local model (Model 5) was only statistically different to Model 1 (mean difference = 0.727, p < 0.05); but not to other models. Moreover, the mean difference between the local model and the pantropical model 2 (Model 2) was the minimal and not statistically significantly different (mean difference =  − 0.210, p > 0.05) from each other. Another observation is that the incorporation of ‘tree height’ parameter in Model 1 and 3 resulted in significant difference from the other 3 models without height. Some studies showed the incorporation of height did not improve AGB predictions, or even reduce the prediction accuracy, due to error propagation in estimating tree height^57,58^. In addition, height estimation can be challenging in closed canopy and the variations of tree shapes, leading to difficulties in locating the tree top accurately^49^.

**Table 6:** The Tukey HSD Post-Hoc analysis: multiple comparisons among the 5 allometric models. Pantropical model 2 found to be not statistically significantly different from the Local model (Model 5).

(I) Model	(J) Model	Mean difference (I–J)	Std. error	Sig.*	95% confidence interval	
					Lower bound	Upper bound
Local [Model 5]	Pantropical 1 [Model 1]	0.727*	0.198	0.002	0.188	1.266
	Pantropical 2 [Model 2]	− 0.210	0.198	0.827	− 0.749	0.330
	Subtropical 1 [Model 3]	0.287	0.198	0.595	− 0.252	0.826
	Subtropical 2 [Model 4]	− 0.379	0.198	0.308	− 0.918	0.160

Lastly, the DBH range and the species coverage in the original study and the applied study were compared. Most of the allometric models tended to exclude the trees with large diameter since they are more challenging to be harvested for measurement. If we apply the model with DBH outside the range, the model tends to underestimate AGB since the trees with higher DBH have larger biomass. Comparing the DBH and species ranges listed in Table 5, the pantropical model 2 was more preferable over the local model in this study.

Pantropical model 2 found to be not statistically significantly different from the Local model (Model 5).

### Stage 2: LiDAR AGB model derivation

The models in various forms (i.e., Model I/II/III in Table 4) were compared and then across the plot-size (i.e., 10 m/5 m/2.5 m radius) to determine the most desirable model. As mentioned above that Model II, using log-transformed dependent variable (i.e., AGB), is more ideal due to its normality. The summary statistics of regression models in various plot sizes of Model II were shown in Table 7. In terms of plot size, the model R^2^, Adjusted R^2^ and RMSE suggested that the plot size in 10 m radius had the highest model explanatory power (R^2^ = 0.864) and lowest RMSE (37.75 kg/ha). The corresponding equations of Model II in the 10 m, 5 m and 2.5 m radius plot size are listed as Eqs. (13)–(15), respectively.

**Table 7:** Summary of model statistics, for the Model II–Log-transformed dependent variable (i.e. AGB) and Raw independent variables, across the 10 m, 5 m, and 2.5 m radius plot-size.

10 m radius				5 m radius				2.5 m radius			
R^2^	Adj. R^2^	MAE*	RMSE (kg/ha)	R^2^	Adj. R^2^	MAE*	RMSE (kg/ha)	R^2^	Adj. R^2^	MAE*	RMSE (kg/ha)
0.864	0.844	0.037	37.75	0.536	0.527	0.044	195.53	0.041	0.036	0.050	1398.32

13 $$\text{ln}AGB=5.961+0.013\left(CCF\right)+0.134\left(P95\right)-0.279(MA{D}_{mode})$$

14 $$\text{ln}AGB=4.546+0.205\left(P50\right)+0.007(CCF)$$

15 $$\text{ln}AGB=2.983+0.004\left(CCA\right)+0.017(In{t}_{P99})$$

**Figure 6:**
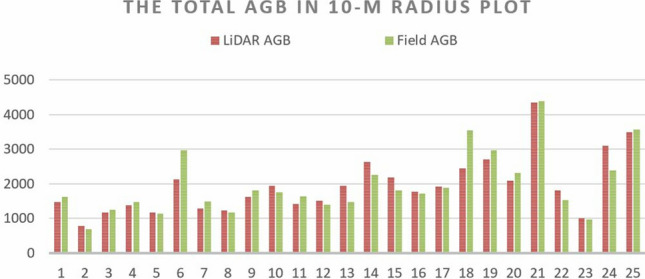
The total AGB computed by Model II (LiDAR AGB) and allometric model 2 (Field AGB) in each 10 m radius plot-level (in kg).

After taking logarithmic transformation, the model R^2^ improved from 0.726 in Model I to 0.864 in Model II of 10 m radius plot (Eq. (13)). This model was built with three predictors in the order of, canopy cover by first returns above 3.66 m (CCF), 95th Height Percentile (P95) and median of absolute deviation from mode (MADmode). Canopy cover explained most of the variance (R^2^ = 0.726), followed by the remaining two predictors under the height and descriptive categories (R^2^ = 0.072 and 0.066 respectively). The dependent variable, log-AGB, increased by 0.013 kg or 0.134 kg when the CCF and P95 increased by 1 percent or 1 m respectively. On the other hand, it decreased by 0.279 kg when the MADmode increased by 1 m (Table 8). Figure 6 shows the comparison between estimation of plot-level AGB by Model II in 10 m radius plot size and the field-measured AGB by Pantropical Model 2. The mean difference between them were minimal and no statistical significant difference had been detected (mean difference = − 23.77, p = 0.753) from the paired t-test.

**Table 8:** The model coefficient and collinearity statistics of Model II—10 m radius plot.

Model	Unstandardized coefficients		Standardized coefficients	Sig.*	Correlations		Collinearity statistics	
	B	Std. Error	Beta		Zero-order	Partial	Tolerance	VIF
(Constant)	5.961	0.144		0.000				
CCF	0.013	0.002	0.567	0.000	0.852	0.755	0.562	1.780
P95	0.134	0.029	0.636	0.000	0.758	0.708	0.339	2.954
MAD model	− 0.279	0.088	− 0.359	0.004	0.282	− 0.571	0.512	1.955

Table 9 summarized the averaged AGB and total AGB in plot-level and scaled up to hectare level computed by Model II. The results indicated that the smaller plot-size tended to underestimate the plot-level AGB within the plot. The field-measured AGB computed by Pantropical Model 2 for the 1 ha study area was 63,341.71 kg, and that of LiDAR-derived Model II, in the plot size of 10 m, 5 m and 2.5 m radius, were 61,912.55 kg, 54,400.66 kg and 34,296.00 kg respectively. It indicated the inverse relationship between the plot size and model accuracy.

**Table 9:** The AGB computed by Model II in various plot sizes.

	Field measured AGB^a^	LiDAR-derived AGB Model		
		10 m radius	5 m radius	2.5 m radius
Total stems	20,625	16,182	15,538	15,100
Avg. stem per plot		647	155	39
Mean (SD) AGB (kg/plot)		1945.04 (826.97)	427.26 (200.10)	67.34(12.17)
Total AGB (kg/plot)		48,626.00	42,726.14	26,936.00
Total AGB (kg/ha)	63,341.71	61,912.55	54,400.66	34,296.00

^a^Computed by the Pantropical Model 2 (Model 2, as shown in Eq. (4)).

### Stage 3: model evaluation and validation

**Figure 7:**
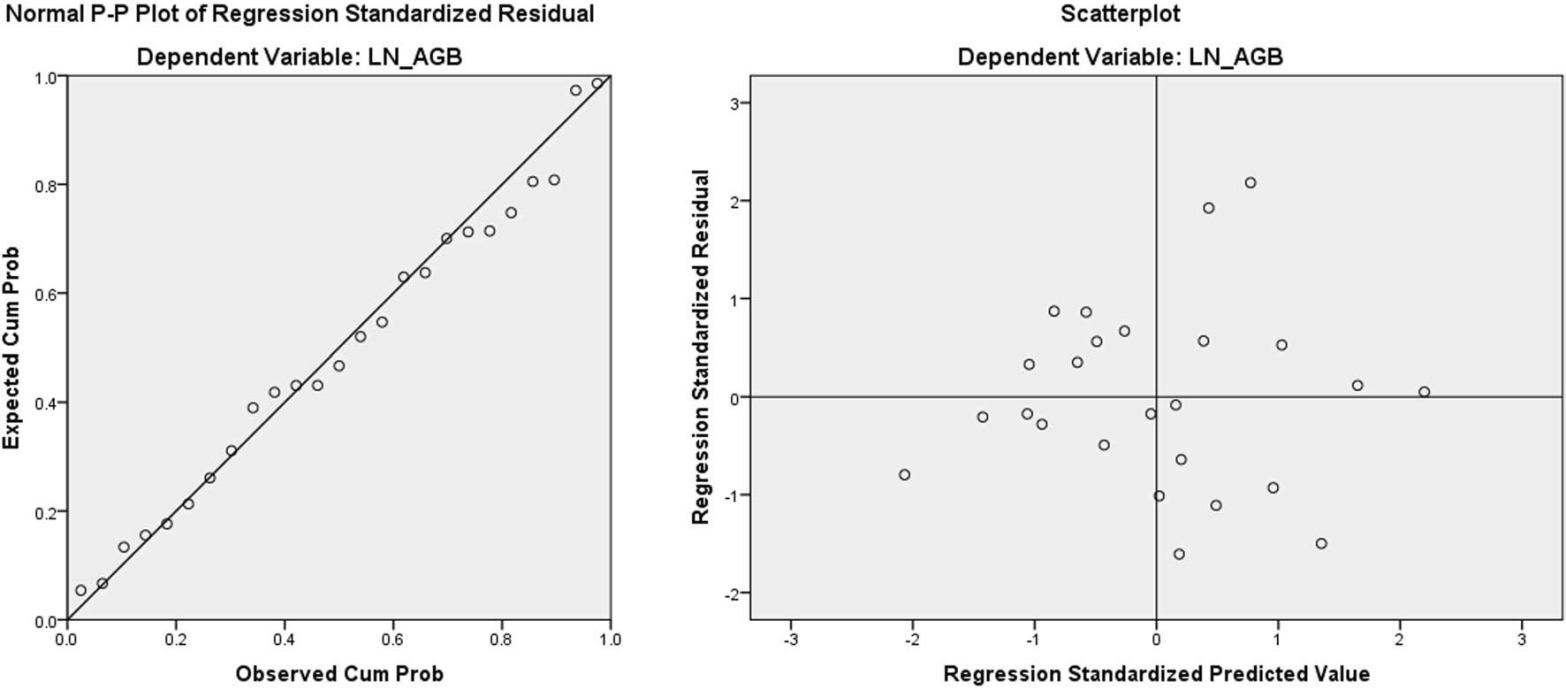
Model II—10 m radius plot model (**a**) residual normal P–P plot and (**b**) residual scatter plots. The results suggested the model residuals were normally distributed and homoscedastic.

The normal P–P plot in Fig. 7a showed that Model II in 10 m radius plot exhibited a normally distributed residuals as the sample plots approximately lied on the reference diagonal line. The homoscedasticity was tested by evaluating the scatter plot of the standardized residual against the standardized predicted value as shown in Fig. 7b. The model residuals were scattered randomly and without any pattern (e.g., cone or fan shape), which indicated the residuals were homoscedastic and variance was equally distributed. Lastly, the multi-collinearity of predictors in the model were checked by the Tolerance and VIF. Referring to the model coefficient table in Table 8, there was no collinearity problem as indicated by the Tolerance value >  > 0.1 (i.e., > 0.3) and VIF <  < 10 (i.e., less than 3).

From the bootstrap table (Table 10), it was confident that all the predictors were significant in predicting the AGB in log scale. The bootstrapping test on the model bias showed that there was minimal level of bias (bias < 0.005). LOOCV was conducted to validate the model. If the predicted R^2^ was found to be significantly smaller than the original model R^2^ (R^2^ of Model II—10 m radius plot was 0.864), the model would then be over-fitting. The reported PRESS statistic was 0.84 and hence predicted R^2^ was 0.81 and CV-RMSE of was 38.88 kg/ha. The RMSE of CV-model was approximated to the RMSE of Model II—10 m radius plot (i.e., 37.75 kg/ha). Therefore, the cross-validated model suggested a reasonable prediction of AGB in 10 m radius plot-level.

**Table 10:** Bootstrapping results of the model predictor of the selected model—Model II—10 m radius plot.

Model predictor	Unstandardized beta coefficient	Bootstrap				
		Bias	Std. error	Sig. (2-tailed)^a^	BCa 95% Confidence	
					Lower	Upper
(Constant)	5.961	− 0.011	0.145	0.001	5.725	6.163
CCF^b^	0.013	0.000	0.003	0.001	0.007	0.017
P95^b^	0.134	0.004	0.035	0.001	0.065	0.254
MADmode^b^	− 0.279	0.004	0.094	0.006	− 0.462	− 0.087

^a^The bootstrap beta coefficient is significant at 0.05 level.

^b^CCF: Canopy cover by first returns above 3.66 m (CCF), 95th Height Percentile (P95) and median of absolute deviation from mode (MADmode). The bootstrapping test on the model bias showed that there was minimal level of bias (bias < 0.005).

## Discussion

### The outperformance of the selected allometric model

There is only one existing allometric model developed specifically in Hong Kong^47,59^. The most ideal and the conventional approach of AGB estimation is to formulate an empirical, site- and species-specific model. Therefore, a locally developed model shall be the most appropriate one potentially. However, the local model was only relied on DBH as the sole predictor on AGB while wood density was suggested to be the second to DBH in terms of AGB predictors^16,60,61^. Therefore, to eliminate the concerns on the applicability of non-local models due to site differences, ANOVA analysis was adopted and found there was no statistical significant difference between three out of the four non-local models, to the local model.

The concern of extrapolation of pantropical allometric models to other regions is the site variations caused by the difference in climatic and vegetation characteristics^14^. In our study, the Pantropical Model 2 developed by Chave et al.^16^ was found to be not statistically different from the local model. The possible reason behind was the inclusion of “Bioclimatic Variable (E)”. The Pantropical Model 1, without the “Bioclimatic variable”, was significantly different from the local model statistically. The inclusion of the bioclimatic variable (i.e., Model 2), which accounted for the temperature and precipitation seasonality, as well as the drought intensity, has greatly reduced the difference. After equalizing the climatic variations, the pantropical model became applicable to the extrapolated area, such as the subtropical forest in Hong Kong. This is supported by some latest studies in Congo Basin and India, which applied the same model equation in estimating AGB with high model accuracy^60,62^. The advantage of using pantropical models, given its statistical applicability, is that the AGB estimated among global forests becoming comparable as they are measured by the same model instead of various local models with their own set of predictors and assumptions.

### Justification of the predictor variables of Model II—10 m radius plot

The correlations between the predictor variables and the dependent variable (i.e., log-AGB) were shown in Table 11. AGB was positively associated with canopy cover (CCF, r = 0.852) and 95th height percentile (P95, r = 0.758). The insignificant positive correlation between MADmode and AGB (r = 0.282, p > 0.05) was possibly due to its strong correlation with P95.

**Table 11:** The Pearson’s correlation among the predictors and dependent variable of the selected Model—Model II 10 m radius plot.

	Log-AGB^a^	CCF	P95	MADmode
Log-AGB	1	0.852**	0.758**	0.282
CCF	0.852**	1	0.650**	0.357
P95	0.758**	0.650**	1	0.689**
MADmode	0.282	0.357	0.689**	1
**Correlation is significant at the 0.01 level (2-tailed). n = 25				
**Partial correlations**				
Log-AGB	1.000	0.755**	0.708**	-0.571**
**Correlation is significant at the 0.01 level (2-tailed). d.f. = 21				

^a^AGB was positively associated with canopy cover (CCF) and 95th height percentile (P95). The insignificant positive relationship with MAD mode with log-AGB due to its strong correlation with P95; as indicated by the significant negative partial correlations between Log-AGB and MAD mode.

The correlation coefficients in Table 11 suggested the plot-level AGB was positively associated with canopy cover (r = 0.852, p < 0.001) and 95th height percentile (r = 0.758, p < 0.001), which inferred vegetation abundance and the canopy height distribution of the plots respectively.

Canopy cover, as indicated by the percentage of first returns above 3.66 m, illustrated the canopy closure by the canopies and particularly the leaves. Broader leaves tended to block the pulse penetration onto the forest ground^51^, resulting in higher proportion of canopy hits to ground hits. The first returns instead of all returns were more promising due to lower chance of mixing the signal with ground returns and thus more capable to explain the biomass content.

Height percentiles were commonly used for AGB estimation since they were highly correlated with various biomass components^31^, yet there was functional difference of lower or upper percentile. Upper percentile was usually used to detect forest growth and better than using maximum height since the latter was less reliable with its low point density^63^. Our model had selected P95, which was upper height percentile, could be due to the canopy that started high above ground. Therefore, AGB was more correlated to the upper percentile, since it was more representative to the biomass components of the study area.

There was an indirect relationship between AGB and MADmode. An insignificant positive correlation with MADmode (r = 0.282, p ≥ 0.05) was observed. The weak positive relationship could possible due to its moderately strong correlation with the previous predictor, P95 (i.e., r = 0.689, p < 0.01) as shown in the Pearson’s correlation table (Table 11). On the other hand, it appeared to be a negative beta coefficient when predicting AGB (See Table 10). It can be explained by the partial correlation between MADmode and log-AGB as shown in Table 11. A significant negative correlation (− 0.571, p < 0.01) was observed. MADmode was fundamentally a neutral predictor that represented the variability or the spread of point cloud distribution. A negative coefficient in MADmode (Eq. (13)) implied a higher AGB was associated with the plot that had small variation in stem height (i.e., smoother and flatter), instead of highly varying (i.e., abrupt and undulating). Moreover, the negative deviation from elevation mode shall result in lower AGB value. However, it turned positive after taking the absolute value by the nature of this parameter (i.e., absolute deviation from mode). To adjust so, the MADmode shall be negatively associated with AGB.

In summary, the canopy cover and 95th height percentile defined the AGB content; since they described the vegetation abundance (or density) and the height of trees respectively. Hence, MAD mode provided a mild adjustment to the model, by considering the degree of spread in vegetation distribution with the plot. Combining the three predictors, the model was capable to depict the forest structure and hence appliable to AGB estimation.

### The effect of plot size on LiDAR-derived model

The LiDAR point density may affect the accuracy of the model, especially when the plot size was getting smaller. The average point density of the LiDAR point cloud was 8.85 per m^2^, which was considered to be moderately high. However, when the plot became smaller; such that there were just around 175 points within each 2.5 m radius plot; and approximately 700 points in each 5 m radius plot. The limited number of returns within the small plot led to deterioration of the accuracy on AGB estimation; especially in such a heterogeneous mixed forest which caused relatively high coefficient of variation within a small plot^59^. Each 10 m radius plot has around 2800 points, which was more capable than the smaller plots in capturing the spatial variation and thus averaging the spatial error of the LiDAR model.

The LiDAR point clouds were clipped by the plot boundary for model building (i.e., within 10 m radius; 5 m radius and 2.5 m radius). On the other hand, the stem location was the determinant of whether that tree would be included or to excluded as a biomass entity during field measurement. If the stems were located outside the plot yet their crowns extended into the plot, then it would be captured by the LiDAR data. Likewise, if the stems were located within the plot yet the crowns extended beyond the plot boundary; that parts of the crowns would not be captured by the LiDAR data. This boundary effect resulted in model errors in terms of model commission or omission^64,65^ which led to over- or under-estimation of the plot-level AGB. For that matter, a larger plot size is recommended since the perimeter-to-area ratio is lower and so the boundary effect. Moreover, the reduction of boundary effect implies lower chance of plot positioning error as there is higher probability of overlapping between the field measurement and LiDAR data plots^66^.

### The role of LiDAR plot metrics on AGB estimation

Conducting field work and measurement on direct harvesting or allometric modeling to estimate AGB for the entire territory is challenging and cost ineffective. The field work conducted for this one hectare plot of this relatively flat and easily accessible study area took nine months. The level of difficulties in carrying out field work for the entire territory, especially on steep slopes, is much higher and time-consuming as it requires extensive manpower effort and the post-analysis work to minimize the slope effect.

LiDAR data is able to quantify the vertical structure of forest, which is an important input to the biomass and carbon estimation, in a relatively cost-effective way. The total flight time of the LiDAR survey that covers the whole territory of Hong Kong (i.e., 1106 km^2^) was just 44 hours. Th promising result from the LiDAR-derived modeling from this study provides an opportunity to enhance the efficiency of terrestrial biomass and carbon pool estimation, especially in the hilly and densely vegetated regions in Hong Kong.

Through generating the relevant metrics from each 10 m radius plot across the territory, The AGB in each hectare of the vegetated area can be estimated with reasonable accuracy. Together with LiDAR technology, it is feasible to estimate AGB in the rugged relief and inaccessible area and hence a more comprehensive terrestrial carbon stock estimation can be facilitated. However, it is noted the LiDAR plot metrics model was developed in the particular dynamic forest plot in Hong Kong, the LiDAR-derived AGB model is more applicable to similar ages and natural subtropical forest. The application of the model to other forest stands (e.g., mature forest) still require further investigation.

## Conclusion

Using a subtropical mixed young forest under the ForestGEO global network as a pioneer testing ground in Hong Kong, this study adopted the integrated methodology approach to assess the feasibility of combining LiDAR plot metrics, allometric equations and field-measured structural variables to estimate AGB. The results suggested that the pantropical allometric model developed by Chave and his collaborators, after the incorporation of the Bioclimatic variable, E, in the allometric model, surpassed a locally developed allometric model and it is applicable to subtropical forest regions. Regression models using the LiDAR plot metrics as model predictors were developed and compared to predict AGB at different plot sizes and the plot-size of 10 m radius performed the best with model R^2^ of 0.864 and RMSE of 37.75 kg/ha. The model selected the predictors which represented the vegetation abundance (i.e., canopy cover by first returns above 3.66 m), tree heights (i.e., 95th height percentile) and variation of stem heights within the plots (median of absolute deviation from mode) respectively. The plot-size of 10 m radius was more superior to the smaller plot size due to the adequate point density and the spatial error was being averaged. Further to this study, the validated model can be used to quantify AGB and carbon stock in the less accessible area with similar forest structure. The estimated AGB provided the basis to the terrestrial biomass and carbon stock baseline map for Hong Kong. The estimation approach can be adopted in subtropical forests with similar forest structure.
